# Prevalence of Overweight and Obesity in Adolescents: A Systematic Review

**DOI:** 10.1155/2013/392747

**Published:** 2013-06-27

**Authors:** Maria del Mar Bibiloni, Antoni Pons, Josep A. Tur

**Affiliations:** ^1^Research Group on Community Nutrition and Oxidative Stress, University of Balearic Islands, 07122 Palma de Mallorca, Spain; ^2^CIBERobn (Fisiopatología de la Obesidad y la Nutrición CB12/03/30038), Instituto de Salud Carlos III, 15706 Santiago de Compostela, Spain

## Abstract

*Objective*. To review the extant literature on the prevalence of overweight and obesity in adolescents (10–19 years old) of both sexes. *Design*. The search was carried out using Medline and Scopus considering articles published from the establishment of the databanks until June 7, 2012. Data on the prevalence of children being overweight and obese from the International Obesity Task Force (IOTF) website was also reviewed. Only original articles and one National Health Report were considered. Forty studies met the inclusion criteria. *Results*. Twenty-five of these studies were nationally representative, and ten countries were represented only by regional data. *Conclusions*. The prevalence of overweight and obesity among adolescents worldwide is high, and obesity is higher among boys. The IOTF criterion is the most frequently used method to classify adolescents as overweighed or obese in public health research.

## 1. Introduction

The prevalence of overweight and obesity among children and adolescents has widely increased worldwide [1, 2], making it one of the most common chronic disorders in this age group and in adulthood. 

The use of body mass index (BMI) for age to define being overweight and obese in children and adolescents is well established for both clinical and public health applications, because of their feasibility under clinical settings and in epidemiological studies [3, 4]. In children and adolescents, the natural increases in BMI that occur with age necessitate the use of age-sex-specific thresholds. The most widely used growth charts are the Centers for Disease Control and Prevention (CDC-2000) [5], the International Task Force (IOTF) [6], and the 2007 growth references for 5 to 19 year olds produced by the World Health Organization (WHO-2007) [7]. 

The CDC-2000 growth charts were developed to evaluate the nutritional status of US children and were originated from five cross-sectional representative surveys carried out in the US between 1963 and 1994. These growth charts are routinely applied to identify children and adolescents with a BMI greater than the 85th or 95th percentiles following the advice of the US Expert Committee on Childhood Obesity [8]. However, the appropriateness of an American dataset for defining overweight in young people from other countries is questionable [9].

The IOTF reference also uses age-sex-specific BMI percentiles, and overweight and obesity definition corresponds to an adult BMI of 25 and 30 kg/m^2^, respectively, and reflects values in children tracking to overweight and obesity in adults [6]. This reference is based on six large international cross-sectional representative datasets, identifying the BMI values that extrapolate to childhood. 

The WHO-2007 growth references were created to replace the National Center for Health Statistics (NCHS) references [10, 11]. This reference was constructed using data from the 1977 NCHS/WHO growth reference (1 to 24 years old) merged with data from the 2006 WHO Child Growth Standards for preschool children (under 5 years of age) using state-of-the-art statistical methods [7].

Although valuable information has been appearing in the literature or online, such as works from the Health Behaviour of School-aged Children study which is mainly related to social determinants of health and well-being among young people [12], no systematic review has been conducted to understand the worldwide magnitude of the overweight and obesity problem among the adolescent population. Thus, the objective of this study was to systematically review the literature regarding the prevalence of overweight and obesity in adolescents (10–19 years old) of both sexes published in the past 12 academic years (1999–2011).

## 2. Methods

**Figure 1:**
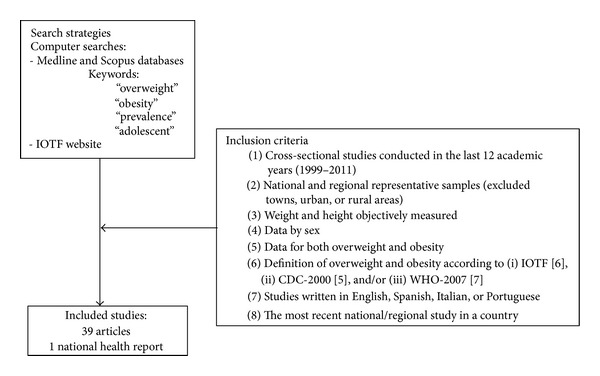
Flow diagram of study selection.

A systematic literature search was performed which ended on June 7, 2012 (see Figure 1). The literature search was conducted in Medline and Scopus using the following MeSH terms: “overweight”; “obesity”; “prevalence”; “adolescent”. In total, 2537 articles were selected. We also reviewed the data on the prevalence of childhood overweight and obesity on the International Obesity Task Force Website at http://www.iaso.org/iotf/. To find the articles included in this review, the following inclusion criteria were used: (1) cross-sectional studies conducted in the last 12 years (1999–2011)—when the original study did not report the survey year, it was not included; (2) national and regional representative samples, but articles published on the prevalence of overweight in towns, urban, or rural areas in a country were excluded; (3) weight and height objectively measured; (4) results presented by sex; (5) and for both overweight and obesity prevalence; (6) the definition of overweight and obesity using the (i) CDC-2000 [5], (ii) IOTF [6], and (iii) WHO-2007 [7] growth references; and (7) studies written in English, Spanish, Italian, or Portuguese. Moreover, if there were more than one national or regional study in the same country, the most recent one was included in the prevalence tables (except for USA [13] and Canada [14], countries in which the most recent data was not included in the tables due to differences in the representativeness of the samples [13] and the impossibility to calculate a single prevalence of overweight and obesity for adolescents' boys and girls [14]; however, no differences in prevalence were observed between studies as it has been indicated in the discussion). The final number of articles included in this review was 39 articles related to overweight and obesity and also a study on the latest statistics on the prevalence of overweight and obesity in South Africa [15].

Potentially relevant papers were selected by (1) screening the titles; (2) screening the abstracts, and (3) if abstracts were not available or did not provide sufficient data, the entire article was retrieved and screened to determine whether it met the inclusion criteria. Full-text articles were assessed by 2 authors (M. M. Bibiloni and J. A. Tur). Any matter of doubt was discussed by at least two of the reviewers (M. M. Bibiloni, A. Pons, and J. A. Tur).

## 3. Results

### 3.1. Literature Search

A total of thirty-nine articles and a National Health Report were eligible according to the inclusion criteria established for this review.  Table 1 presents a description of the forty studies selected for this review including the continent and the country where it was performed (and region for not national studies), year of publishing, total number of participants in the study, number of adolescents, age range, proportion of girls, and number and definition for overweight and obesity classification used. All the articles were published after the year of 2002. Nationally representative data were obtained in twenty-five countries (including Northern Ireland) [15–39], and ten countries were represented only by regional data [40, 42, 44, 45, 47, 50–54].

### 3.2. Prevalence and Criteria for Classification

Table 2 shows overweight and obesity prevalence from the twenty-five national studies (one of them including data from Northern Ireland) that were included in this review according to the continent and the country where it was performed, year of survey, study population, age range, criteria used for classifying overweight and obesity used, and along with total data by sex. There were thirty-two different prevalence levels described in the included articles, because five countries presented data using at least two different criteria for overweight and obesity classification [18, 23, 27, 36, 39]. The IOTF cut-off was used to classify overweight and obesity in twenty-three of the twenty-five national studies considered in the present review.

There was a broad range of overweight and obesity prevalence noted. In general, the prevalence of overweight plus obesity was higher in America [18–20], Oceania [38, 39] and Europe [30–37] and lower in Africa [15–17] and certain parts of Asia [21–29] (in China [22] and Iran [23] the total prevalence was less than 10% by the IOTF cut-offs). Overall, about 30% of American adolescents and 22%–25% of European adolescents (excepting the Czech Republic and Italian adolescents' which showed a prevalence of 13.7% and 17.9%, resp.) were overweight or obese. Among Oceanian adolescents the prevalence ranged from 23.2% in Australia in 2004 to 34.2% in New Zealand in 2007. In Africa, the overall prevalence of overweight and obesity was lower than 20%. Among Asian adolescents there was a broad range of overweight plus obesity. Using IOTF cut-off, the prevalence of being overweight or obese for Asian boys and girls ranged from 5.2% in China in 2002 to 36.4% in Bahrain in 2000.

Table 3 shows regional data prevalence of overweight and obesity from fifteen countries. Specific prevalence from all the geographic regions was included in this review from three countries: South Africa (nine provinces) [15], USA (fifty two states) [20], and Italy (five regions) [34]. In Europe, data from islands of Greece (Crete) [46] and Italy (Sicily and Sardinia) [48, 49] and Spain (Balearic Islands' archipelago [51]; and the Grand Canary Island [52]) were also included. On the other hand, regional but not national data was found for eleven countries (Italy [34], Brazil [40], India [42], Jordan [43], Denmark [44], France [45], Hungary [47], Poland [48], Spain [51, 52], Switzerland [53], and Turkey [54]). The IOTF cut-off was used to classify overweight and obesity in fourteen of the eighteen selected studies that included regional data. In one study [51], data was presented using only the WHO-2007 growth charts and in two studies using only the CDC-2000 growth reference [20, 43].

In South Africa and USA, substantial geographic variations in adolescent overweight and obesity existed. In 2008, overweight and obesity prevalence varied in South Africa from 13.5% in Limpopo to 25.5% in KwaZulu-Natal. In 2007, overweight and obesity varied in USA from 23.1% in Utah and Minnesota to 44.5% in Mississippi. In 2002, the prevalence of overweight and obesity in Southern Italy and Italian islands was higher among boys. In Southern Italy, the overweight prevalence among girls also was higher than in the other geographic regions.

Comparison between the islands from Greece (Crete), Italy (Sicily and Sardinia), and Spain (Balearic Islands and Grand Canary Island) which were included in this review showed that Crete had the highest prevalence of overweight and obesity—despite data were presented using different definition. In Spain, using the IOTF cut-off (data not shown for Balearic Islands but given by authors), the prevalence of overweight plus obesity was higher in the Grand Canary Island (29.1%) than in the Balearic Islands (24.7%). 

### 3.3. Gender Differences

According to national data, the prevalence of overweight among boys was ≥10% higher than girls in nine countries (Canada [18], Qatar [26], Taiwan [28], Cyprus [30], Czech Republic [31], Germany [32], Greece [33], Italy [34], Australia [38], Denmark [44], and Hungary [47]) and among girls ≥10% higher than boys in seven of the twenty-five countries (South Africa [15], Seychelles [16], Tunisia [17], Mexico [19], Bahrain [21], Saudi Arabia [27], and Sweden [37]). The obesity prevalence was ≥10% higher among boys in seventeen countries (Canada [18], USA [20], China [22], Iran [23], Israel [24], Qatar [26], Saudi Arabia [27], Taiwan [28], Cyprus [30], Czech Republic [31], Germany [32], Greece [33], Italy [34], Portugal [36], Sweden [37], Australia [38], New Zealand [39], Denmark [44], and Hungary [47]) and ≥10% higher among girls in four of the twenty-five countries (South Africa [14], Seychelles [16], Tunisia [17], and Bahrain [21]).

## 4. Discussion

The aim of this study was to review systematically the literature on overweight and obesity prevalence among adolescents worldwide. Thirty-nine articles and one National Health Report that met the inclusion criteria were considered. The overweight and obesity prevalence in the included studies ranged widely. In sixteen of the twenty-three countries with national representative data using the IOTF cut-off, overweight and obesity prevalence higher than 20% were found, five countries showed prevalence above 30%, and just in two countries prevalence was lower than 10%.

Regarding national data, when prevalence was analysed according to sex, it was observed that boys showed a higher prevalence of overweight in almost half of the countries and a higher prevalence of obesity in almost all countries. These results are consistent with previous studies that pointed out a high prevalence of abdominal obesity among boys [55]. Differences of prevalence of overweight and obesity between genders have been related to geopolitical and cultural conditions [55].

Eight articles compared data between 1980s and/or 1990s with 2000s [16, 19, 20, 22, 28, 32, 37, 50] and pointed out an increased prevalence of overweight and obesity in both sexes over this period. However, among Australian adolescents [38] the overweight and obesity prevalence increased significantly among boys but not among girls over the period 1997–2004. In the Australian National Children's Nutrition and Physical Activity Survey 2007 (NCNPAS07) [14], 25% of boys and 30% of girls aged 9- to 13-year-olds and 25% of boys and 23% of girls aged 14- to 16-year-olds were overweight or obese using the IOTF criteria. A comparison of the 1985, 1995, and 2007 Australian national surveys of 7- to 15-year-olds indicated that Australian children are changing body shape to a more central fat distribution [14]. In USA, overweight and obesity prevalence among adolescents increased 4% in 2003 and 10% in 2007. Overweight and obesity prevalence increased by 3% and 18% among USA girls over this period. However, a cross-sectional analyses of a representative sample (*n* = 4111) of the USA child and adolescent population (birth through 19 years of age) with data from the National Health and Nutrition Examination Survey 2009-10 (NHANES) indicated a prevalence of overweight and obesity among adolescents aged 12 through 19 years of 15.2% and 18.4%, respectively. Analyses of trends in obesity prevalence for the last two NHANES surveys (2007-08 and 2009-10) indicated that the prevalence of obesity in children and adolescents has not changed in 2009-10 compared with 2007-08 [13]. On the other hand, since 2004 the overweight and obesity trends were stabilized or decreased among German adolescents [32].

In USA, substantial geographic disparities in adolescent overweight and obesity were found, with an apparent shift toward higher prevalence in 2007 for several states [20]. Generally, overweight and obesity prevalence was also higher in southern USA in 2007. Lobstein et al. [56] reported that children in Northern Europe countries generally tended to have lower overweight and obesity prevalence (10–20%) than in Southern Europe (20–35%). Also within the same country, the prevalence and trends of overweight and obesity may not be homogeneous according to different geographic regions [57]. In Italy, a north-south gradient in overweight and obesity prevalence among boys but also in overweight prevalence among girls was also reported [34]. A higher prevalence of overweight and obesity has been reported in Southern Spain in both children [58] and adults [59].

It is important to note that the choice of a reference and a cut-off point will determine the absolute prevalence of overweight and obesity and its trends, and hence different information will be obtained from the papers [60]. The IOTF classification for adolescent overweight and obesity [6] is the most frequently used. Cole et al. [6] argued that the reference they published, supported by the IOTF, is less arbitrary and more international than others and recommended its use in international comparisons. Lately, Monasta et al. [61] suggested that the IOTF reference and cut-offs could be preferable to identify overweight and obesity both at individual and population levels because they are at least based on a crude association with ill and health later in life, namely, the definition of overweight and obesity at age 18 years. However, the IOTF cut-offs have been not recommended for clinical use when assessing an individual child's growth [9, 62–64]. Furthermore, recent findings suggested that a universal BMI classification system for childhood and adolescent overweight and obesity may not correspond to a comparable level of body fatness in all populations [9]. The prevalence estimates may not accurately characterize the population groups most at risk of health disadvantages because the correlation of BMI with adiposity is highly variable and dependent on ethnic group [9, 60, 65, 66].

## 5. Limitations of the Study

The comparisons of overweight and obesity prevalence need interpretation with caution due to the difference in survey sampling methods, sample sizes, age range of subjects, quality of data in terms of height and weight measurement, and whether national programmes or strategies to tackle overweight and obesity are in place [57]. Even within the same country, the prevalence and trends of overweight and obesity may not be homogenous in view of different ethnicities, geographic regions, and socioeconomic status [57]. Only articles in English, Spanish, Italian, and Portuguese were included in this review.

## 6. Conclusions

The results of this review allow the following conclusions: (1) overweight and obesity prevalence is high; (2) obesity is higher among boys, although it is not clear which sex has a higher proportion of adolescents with overweight; (3) despite that there is no consensus about criteria to be used to classify adolescents as overweighed or obese, the most frequently used was the IOTF reference [6]. However, the international reference charts for monitoring the secular trends in childhood obesity need to be continually refined and evaluated [56]. The results of this study would contribute to guiding health planners and administrators to develop proper tools for adolescent obesity management.

## Figures and Tables

**Table 1:** Descriptive analysis of the studies reviewed.

Area	Continent	Country, region	Date of survey	Total *n* of study^1^	Total *n* of adolescents^1^	Age (years)/school grade	Proportion of girls	Number of definition	Definition	Reference
National	Africa	Seychelles	2004	4,854	2,177	7th, 10th	51.5%	1	IOTF	Bovet et al., 2006 [16]
		South Africa	2008	9,862	9,862	13–19	50.9%	1	IOTF	Reddy et al., 2010 [15]
		Tunisia	2004	2,872	2,872	15–19	54.9%	1	IOTF	Aounallah-Skhiri et al., 2008 [17]
	America	Canada	2004	8,661	4,099	12–17	—	3	IOTF, CDC, WHO	Shields and Tremblay, 2010 [18]
		Mexico	2006	48,304	13,219	12–18	50.7%	1	IOTF	Bonvecchio et al., 2009 [19]
		USA	2007	44,101	44,101	10–17	—	1	CDC	Singh et al., 2010 [20]
	Asia	Bahrain	2000	506	506	12–17	50.8%	1	IOTF	Al-Sendi et al., 2003 [21]
		China	2002	44,880	12,475	13–17	47.7%	1	IOTF	Li et al., 2008 [22]
		Iran	2003-04	21,111	16,035	10–18	51.3%	2	IOTF, CDC	Kelishadi et al., 2008 [23]
		Israel	2003-04	5,588	5,588	11–19	55.1%	1	CDC	Nitzan Kaluski et al., 2009 [24]
		Jordan	2009	5,640	637	13–18	55.7%	1	IOTF	Khader et al., 2011 [25]
		Qatar	2003-04	3,923	3,923	12–17	49.8%	1	IOTF	Bener, 2006 [26]
		Saudi Arabia	2005	19,317	7,251	13–18	49.2%	2	CDC, WHO	El Mouzan et al., 2010 [27]
		Taiwan	2003	72,789	58,424	10–18	49.0%	1	IOTF	Liou et al., 2009 [28]
		United Arab Emirates	2009-10	1,007	276	11–18	—	1	IOTF	Ng et al., 2011 [29]
	Europe	Cyprus	1999-2000	2,467	1,694	10–17	50.7%	1	IOTF	Savva et al., 2002 [30]
		Czech Republic	2005	1,417	957	11–17	49.4%	1	IOTF	Kunesova et al., 2007 [31]
		Germany	2008	40,622	5,623	12–16	46.7%	1	IOTF	Blüher et al., 2011 [32]
		Greece	2003	14,456	14,456	13–19	53.8%	1	IOTF	Tzotzas et al., 2008 [33]
		Italy	2002	4,386	4,386	11, 13, 15	51.6%	1	IOTF	Vieno et al., 2005 [34]
		Republic of Ireland	2003	17,499	7,294	11–16	50.6%	1	IOTF	Whelton et al., 2007 [35]
		Northern Ireland	2003	2,039	964	11–15	51.5%	1	IOTF	Whelton et al., 2007 [35]
		Portugal	2008	22,048	22,048	10–18	51.5%	2	IOTF, WHO	Sardinha et al., 2011 [36]
		Sweden	2001	1,732	1,732	10, 13, 16	48.3%	1	IOTF	Ekblom et al., 2004 [37]
	Oceania	Australia	2004	5,407	1,771	8th, 10th	45.6%	1	IOTF	Booth et al., 2007 [38]
		New Zealand	2007	8,796	8,796	13–17	45.4%	2	IOTF, WHO	Utter et al., 2010 [39]

Regional	Africa	South Africa, Eastern Cape	2008	926	926	13–19	52.1%	1	IOTF	Reddy et al., 2010 [15]
		South Africa, Free State	2008	1,236	1,236	13–19	49.1%	1	IOTF	Reddy et al., 2010 [15]
		South Africa, Gauteng	2008	931	931	13–19	52.1%	1	IOTF	Reddy et al., 2010 [15]
		South Africa, KwaZulu-Natal	2008	910	910	13–19	52.1%	1	IOTF	Reddy et al., 2010 [15]
		South Africa, Limpopo	2008	1,140	1,140	13–19	50.5%	1	IOTF	Reddy et al., 2010 [15]
		South Africa, Mpumalanga	2008	1,238	1,238	13–19	49.8%	1	IOTF	Reddy et al., 2010 [15]
		South Africa, Northern Cape	2008	1,088	1,088	13–19	48.6%	1	IOTF	Reddy et al., 2010 [15]
		South Africa, North West	2008	1,234	1,234	13–19	48.6%	1	IOTF	Reddy et al., 2010 [15]
		South Africa, Western Cape	2008	1,159	1,159	13–19	56.4%	1	IOTF	Reddy et al., 2010 [15]
	America	USA, 52 Sates^2^	2007	—	—	10–17	—	1	CDC	Singh et al., 2010 [20]
		Brazil, Pernambuco State	2006	4,210	4,210	14–19	59.8%	1	IOTF	Tassitano et al., 2009 [40]
	Asia	China, Hong Kong	2003-04	2,098	2,098	11–18	53.2%	2	IOTF, CDC	Ko et al., 2008 [41]
		India, Manipur	2005-06	3,356	3,356	12–19	56.2%	1	IOTF	Bishwalata et al., 2010 [42]
		Jordan, Irbid Governorate	2007	1,355	1,355	13–16	55.6%	1	CDC	Abu Baker and Daradkeh, 2010 [43]
	Europe	Denmark, Greater Copenhagen area and 3 municipalities outside the Capital Region	2007–09	7,541	7,541	14–16	50.1%	1	IOTF	Søren and Jo, 2010 [44]
		France, Aquitaine Region	2004-05	2,385	2,385	11–18	49.1%	1	IOTF	Thibault et al., 2010 [45]
		Greece, Crete	2005-06	481	481	10–12	54.0%	1	IOTF	Manios et al., 2011 [46]
		Hungary, Szeged and Szolnok regions	2005-2006	14,290	14,290	11–16	48.1%	1	IOTF	Baráth et al., 2010 [47]
		Italy, 5 residence regions^3^	2002	4,386	4,386	11–15	51.6%	1	IOTF	Vieno et al., 2005 [34]
		Italy, Sardinia	1999–2001	3,946	3,946	11–15	49.0%	1	IOTF	Velluzzi et al., 2007 [48]
		Italy, Sicily	1999–2001	48,897	48,897	11–15	50.7%	1	CDC	Baratta et al., 2006 [49]
		Poland, Kujawsko-Pomorskie	2005	—	—	13–15	—	1	IOTF	Jodkowska et al., 2010 [50]
		Poland, Lubuskie	2005	—	—	13–15	—	1	IOTF	Jodkowska et al., 2010 [50]
		Poland, Malapolskie	2005	—	—	13–15	—	1	IOTF	Jodkowska et al., 2010 [50]
		Poland, Podlaskie	2005	—	—	13–15	—	1	IOTF	Jodkowska et al., 2010 [50]
		Poland, Pomorskie	2005	—	—	13–15	—	1	IOTF	Jodkowska et al., 2010 [50]
		Spain, Balearic Islands	2007-08	1,231	1,231	12–17	53.4%	1	WHO	Bibiloni et al., 2010 [51]
		Spain, Grand Canary	2004-05	1,002	1,002	12–14	50.0%	1	IOTF	Henríquez Sánchez et al., 2008 [52]
		Switzerland, Canton of Vaud	2005-06	5,207	5,207	10–14	49.7%	2	IOTF, CDC	Lasserre et al., 2007 [53]
		Turkey, Edirne Province	2001	989	989	12–17	48.1%	1	IOTF	Öner et al., 2004 [54]

^1^Only subjects with anthropometric measurements were included in each country.

^2^There are 52 states in the USA, but no information about total number of subjects was included in each state.

^3^Vieno et al. [34] assessed the overall overweight and obesity prevalence among Italian adolescents, and also according to the geographic region: North-West, North-East, Center, South, and Islands, but no information about total number of subjects was included in each region.

IOTF: International Obesity Task Force; CDC: Center for Disease Control and Prevention; WHO: World Health Organization.

**Table 2:** Description of overweight and obesity prevalence (%) along with total data by sex from each national study that was included in the review according to year of survey, study population, age range, and classification criteria used.

Continent	Country	Date of survey	Study population	Age (years)/school grade	Criteria	Overweight (%)			Obesity (%)			Reference
						All	Boys	Girls	All	Boys	Girls	
Africa	Seychelles	2004	School-based survey	7th, 10th	IOTF^1^	12.0	9.5	14.3	5.1	4.2	6.0	Bovet et al., 2006 [16]
	South Africa	2008	2008 SA YRBS	13–19	IOTF^1^	14.4	7.9	20.6	5.3	3.3	7.2	Reddy et al., 2010 [15]
	Tunisia	2004	Household-based survey	15–19	IOTF^1^	12.4	11.0	14.1	2.6	1.9	3.2	Aounallah-Skhiri et al., 2008 [17]

America	Canada	2004	2004 CCHS	12–17	IOTF^1^	19.8	21.2	18.4	9.4	11.1	7.4	Shields and Tremblay, 2010 [18]
					CDC-2000^2^	15.9	17.0	14.7	12.1	14.3	9.6	
					WHO-2007^3^	20.8	21.9	19.6	12.4	15.1	9.4	
	Mexico	2006	Household-based survey	12–18	IOTF^1^	21.2	20.1	22.3	8.9	9.2	8.6	Bonvecchio et al., 2009 [19]
	USA	2007	2007 NSCH	10–17	CDC-2000^2^	15.2	15.3	15.2	16.4	19.2	13.5	Singh et al., 2010 [20]

Asia	Bahrain	2000	School-based survey	12–17	IOTF^1^	20.0	15.3	24.5	16.4	14.9	17.9	Al-Sendi et al., 2003 [21]
	China	2002	2002 CNNHS	13–17	IOTF^1^	4.6	4.6	4.6	0.6	0.7	0.5	Li et al., 2008 [22]
	Iran	2003-04	CASPIAN Study	10–18	IOTF^1^	5.9	5.7	6.0	1.3	1.5	1.1	Kelishadi et al., 2008 [23]
					CDC-2000^2^	4.5	4.3	4.7	1.9	2.3	1.6	
	Israel	2003-04	MABAT Youth Survey	11–19	CDC-2000^2^	12.9	12.7	13.0	5.6	7.4	4.1	Nitzan Kaluski et al., 2009 [24]
	Jordan	2009	Household-based survey	13–18	IOTF^1^	13.7	11.3	15.5	10.0	12.4	8.2	Khader et al., 2011 [25]
	Qatar	2003-04	School-based survey	12–17	IOTF^1^	23.8	28.6	18.9	6.3	7.9	4.7	Bener, 2006 [26]
	Saudi Arabia	2005	Household-based survey	13–18	CDC-2000^2^	17.9	16.5	19.6	7.0	8.2	5.5	El Mouzan et al., 2010 [27]
					WHO-2007^3^	16.0	13.6	18.4	10.6	11.2	10.0	
	Taiwan	2003	School-based survey	10–18	IOTF^1^	16.3	18.4	14.2	6.2	8.1	4.2	Liou et al., 2009 [28]
	United Arab Emirates	2009-10	Household-based survey	11–18	IOTF^1^	—	16.2	20.5	—	11.7	19.7	Ng et al., 2011 [29]

Europe	Cyprus	1999-00	School-based survey	10–17	IOTF^1^	18.9	21.3	16.5	5.8	7.1	4.5	Savva et al., 2002 [30]
	Czech Republic	2005	Lifestyle and Obesity Study	6–17	IOTF^1^	12.3	16.6	8.0	1.4	1.7	1.0	Kunesova et al., 2007 [31]
	Germany	2008	CrescNet database	12–16	IOTF^1^	18.2	19.3	17.0	6.2	7.6	4.6	Blüher et al., 2011 [32]
	Greece	2003	School-based survey	13–19	IOTF^1^	18.3	23.3	14.0	4.3	6.1	2.7	Tzotzas et al., 2008 [33]
	Italy	2002	HBSC Study	11, 13, 15	IOTF^1^	15.6	20.9	10.6	2.3	3.5	1.2	Vieno et al., 2005 [34]
	Republic of Ireland	2003	School-based survey	11–16	IOTF^1^	18.5	17.8	19.2	5.8	5.6	6.1	Whelton et al., 2007 [35]
	Northern Ireland	2003	School-based survey	11–15	IOTF^1^	18.2	18.5	17.8	5.9	6.0	5.7	Whelton et al., 2007 [35]
	Portugal	2008	School-based survey	10–18	IOTF^1^	17.4	17.7	17.0	5.2	5.8	4.6	Sardinha et al., 2011 [36]
					WHO-2007	21.8	20.4	23.1	9.9	10.3	9.6	
	Sweden	2001	School-based survey	10, 13, 16	IOTF^1^	15.8	14.6	16.9	4.4	5.0	3.6	Ekblom et al., 2004 [37]

Oceania	Australia	2004	2004 SPANS	8th, 10th	IOTF^1^	17.9	19.4	16.2	5.3	6.7	3.6	Booth et al., 2007 [38]
	New Zealand	2007	Youth'07 Survey	13–17	IOTF^1^	24.0	23.3	24.7	10.2	10.8	9.5	Utter et al., 2010 [39]
					WHO-2007	25.9	25.9	26.0	13.5	14.6	12.1	

^1^Overweight and obesity, all adolescents with BMI-for-age ≥25 kg/m^2^ and <30 kg/m^2^ and ≥30 kg/m^2^, respectively, according to the IOTF [6].

^2^Overweight and obesity, all adolescents with BMI-for-age ≥P85th and <P95th and ≥P95th, respectively, according to the CDC [5].

^3^Overweight and obesity, all adolescents with BMI-for-age >+1SD and <+2SD and >+2SD, respectively, according to the WHO [7].

IOTF: International Obesity Task Force; CDC: Center for Disease Control and Prevention; WHO: World Health Organization; 2008 SA YRBS: 2008 South African National Youth Risk Behaviour; 2004 CCHS: 2004 Canadian Community Health Survey; 2007 NSCH: National Survey of Children's Health; 2002 CNNHS: 2002 China National Nutrition and Health Survey; CASPIAN Study: Childhood and Adolescence Surveillance and Prevention of Adult Non-communicable disease; HBSC: Health Behaviour in School-aged Children; 2004 SPANS: 2004 NSW Schools Physical Activity and Nutrition Survey.

**Table 3:** Description of overweight and obesity prevalence (%) along with total data by sex from each regional study that was included in the review according to year of survey, study population, age range, and classification criteria used.

Continent	Country, region	Date of survey	Study population	Age (years)	Criteria	Overweight (%)			Obesity (%)			Reference
						All	Boys	Girls	All	Boys	Girls	
Africa	South Africa, Eastern Cape	2008	2008 SA YRBS	13–19	IOTF^1^	13.3	4.3	21.1	4.0	2.0	5.6	Reddy et al., 2010 [15]
	South Africa, Free State	2008	2008 SA YRBS	13–19	IOTF^1^	11.6	8.1	15.1	4.7	3.7	5.7	Reddy et al., 2010 [15]
	South Africa, Gauteng	2008	2008 SA YRBS	13–19	IOTF^1^	12.7	10.0	15.4	9.7	8.4	11.0	Reddy et al., 2010 [15]
	South Africa, KwaZulu-Natal	2008	2008 SA YRBS	13–19	IOTF^1^	20.1	8.6	31.5	5.4	3.4	7.3	Reddy et al., 2010 [15]
	South Africa, Limpopo	2008	2008 SA YRBS	13–19	IOTF^1^	10.7	6.2	15.1	2.8	1.0	4.5	Reddy et al., 2010 [15]
	South Africa, Mpumalanga	2008	2008 SA YRBS	13–19	IOTF^1^	15.5	10.0	21.1	6.1	2.3	9.9	Reddy et al., 2010 [15]
	South Africa, Northern Cape	2008	2008 SA YRBS	13–19	IOTF^1^	12.9	7.3	18.3	5.0	4.4	5.6	Reddy et al., 2010 [15]
	South Africa, North West	2008	2008 SA YRBS	13–19	IOTF^1^	11.8	7.0	16.7	3.9	2.2	5.7	Reddy et al., 2010 [15]
	South Africa, Western Cape	2008	2008 SA YRBS	13–19	IOTF^1^	14.3	9.7	18.5	5.6	2.0	8.9	Reddy et al., 2010 [15]

America	USA, Alaska	2007	2007 NSCH	10–17	CDC-2000^2^	19.8	22.7	16.7	14.1	14.6	13.7	Singh et al., 2010 [20]
	USA, Alabama	2007	2007 NSCH	10–17	CDC-2000^2^	18.2	17.6	18.9	17.9	24.4	10.9	Singh et al., 2010 [20]
	USA, Arkansas	2007	2007 NSCH	10–17	CDC-2000^2^	17.1	15.0	19.2	20.4	27.2	13.2	Singh et al., 2010 [20]
	USA, Arizona	2007	2007 NSCH	10–17	CDC-2000^2^	12.8	12.7	12.7	17.8	20.6	15.0	Singh et al., 2010 [20]
	USA, California	2007	2007 NSCH	10–17	CDC-2000^2^	15.5	13.4	17.5	15.0	17.4	12.8	Singh et al., 2010 [20]
	USA, Colorado	2007	2007 NSCH	10–17	CDC-2000^2^	13.0	17.5	8.5	14.2	17.5	10.7	Singh et al., 2010 [20]
	USA, Connecticut	2007	2007 NSCH	10–17	CDC-2000^2^	13.2	14.7	11.7	12.5	14.8	10.2	Singh et al., 2010 [20]
	USA, Washington, DC	2007	2007 NSCH	10–17	CDC-2000^2^	15.2	11.6	18.7	20.2	22.2	18.2	Singh et al., 2010 [20]
	USA, Delaware	2007	2007 NSCH	10–17	CDC-2000^2^	19.9	22.0	17.8	13.3	12.2	14.4	Singh et al., 2010 [20]
	USA, Florida	2007	2007 NSCH	10–17	CDC-2000^2^	14.8	12.7	17.1	18.3	21.5	15.0	Singh et al., 2010 [20]
	USA, Georgia	2007	2007 NSCH	10–17	CDC-2000^2^	16.0	14.4	17.7	21.3	24.7	17.7	Singh et al., 2010 [20]
	USA, Hawaii	2007	2007 NSCH	10–17	CDC-2000^2^	17.3	17.5	17.1	11.2	15.0	7.1	Singh et al., 2010 [20]
	USA, Iowa	2007	2007 NSCH	10–17	CDC-2000^2^	15.3	14.8	15.9	11.2	11.3	11.0	Singh et al., 2010 [20]
	USA, Idaho	2007	2007 NSCH	10–17	CDC-2000^2^	15.7	14.4	17.2	11.8	16.4	6.8	Singh et al., 2010 [20]
	USA, Illinois	2007	2007 NSCH	10–17	CDC-2000^2^	14.2	12.1	16.4	20.7	25.0	16.3	Singh et al., 2010 [20]
	USA, Indiana	2007	2007 NSCH	10–17	CDC-2000^2^	15.2	11.5	19.3	14.7	17.4	11.7	Singh et al., 2010 [20]
	USA, Kansas	2007	2007 NSCH	10–17	CDC-2000^2^	14.9	16.0	13.7	16.2	16.2	16.3	Singh et al., 2010 [20]
	USA, Kentucky	2007	2007 NSCH	10–17	CDC-2000^2^	16.1	17.7	14.6	21.0	22.5	19.4	Singh et al., 2010 [20]
	USA, Louisiana	2007	2007 NSCH	10–17	CDC-2000^2^	15.2	15.5	14.9	20.7	23.1	18.1	Singh et al., 2010 [20]
	USA, Massachusetts	2007	2007 NSCH	10–17	CDC-2000^2^	16.7	18.2	15.4	13.3	16.1	10.5	Singh et al., 2010 [20]
	USA, Maryland	2007	2007 NSCH	10–17	CDC-2000^2^	15.2	19.1	11.1	13.6	17.0	9.9	Singh et al., 2010 [20]
	USA, Maine	2007	2007 NSCH	10–17	CDC-2000^2^	15.3	15.6	14.9	12.9	16.0	9.8	Singh et al., 2010 [20]
	USA, Michigan	2007	2007 NSCH	10–17	CDC-2000^2^	18.1	20.4	15.8	12.5	14.3	10.5	Singh et al., 2010 [20]
	USA, Minnesota	2007	2007 NSCH	10–17	CDC-2000^2^	12.0	12.1	11.8	11.1	14.3	7.6	Singh et al., 2010 [20]
	USA, Missouri	2007	2007 NSCH	10–17	CDC-2000^2^	17.4	16.9	17.8	13.6	15.5	11.6	Singh et al., 2010 [20]
	USA, Mississippi	2007	2007 NSCH	10–17	CDC-2000^2^	22.6	21.6	23.5	21.9	25.5	18.5	Singh et al., 2010 [20]
	USA, Montana	2007	2007 NSCH	10–17	CDC-2000^2^	13.8	15.0	12.5	11.8	16.6	6.6	Singh et al., 2010 [20]
	USA, North Carolina	2007	2007 NSCH	10–17	CDC-2000^2^	14.9	13.9	16.0	18.6	19.3	17.9	Singh et al., 2010 [20]
	USA, North Dakota	2007	2007 NSCH	10–17	CDC-2000^2^	14.3	16.9	11.6	11.4	15.7	7.0	Singh et al., 2010 [20]
	USA, Nebraska	2007	2007 NSCH	10–17	CDC-2000^2^	15.7	13.9	17.5	15.8	23.0	8.1	Singh et al., 2010 [20]
	USA, New Hampshire	2007	2007 NSCH	10–17	CDC-2000^2^	16.6	17.1	16.2	12.8	16.3	8.8	Singh et al., 2010 [20]
	USA, New Jersey	2007	2007 NSCH	10–17	CDC-2000^2^	15.6	17.3	13.8	15.4	18.6	11.7	Singh et al., 2010 [20]
	USA, New Mexico	2007	2007 NSCH	10–17	CDC-2000^2^	16.7	15.3	18.1	16.0	20.4	11.4	Singh et al., 2010 [20]
	USA, Nevada	2007	2007 NSCH	10–17	CDC-2000^2^	19.0	21.9	16.0	15.2	19.4	10.8	Singh et al., 2010 [20]
	USA, New York	2007	2007 NSCH	10–17	CDC-2000^2^	15.8	15.1	16.5	17.1	20.3	13.8	Singh et al., 2010 [20]
	USA, Ohio	2007	2007 NSCH	10–17	CDC-2000^2^	14.8	18.7	10.9	18.5	22.9	14.2	Singh et al., 2010 [20]
	USA, Oklahoma	2007	2007 NSCH	10–17	CDC-2000^2^	13.2	18.1	8.2	16.4	17.4	15.4	Singh et al., 2010 [20]
	USA, Oregon	2007	2007 NSCH	10–17	CDC-2000^2^	14.7	16.2	13.3	9.6	11.0	8.2	Singh et al., 2010 [20]
	USA, Pennsylvania	2007	2007 NSCH	10–17	CDC-2000^2^	14.7	15.5	13.7	15.0	21.0	8.4	Singh et al., 2010 [20]
	USA, Rhode Island	2007	2007 NSCH	10–17	CDC-2000^2^	15.7	15.5	16.0	14.4	18.2	10.5	Singh et al., 2010 [20]
	USA, South Carolina	2007	2007 NSCH	10–17	CDC-2000^2^	18.5	20.8	15.9	15.3	18.4	12.0	Singh et al., 2010 [20]
	USA, South Dakota	2007	2007 NSCH	10–17	CDC-2000^2^	15.2	17.3	13.0	13.2	16.0	10.2	Singh et al., 2010 [20]
	USA, Tennessee	2007	2007 NSCH	10–17	CDC-2000^2^	15.9	14.5	17.3	20.6	23.3	17.9	Singh et al., 2010 [20]
	USA, Texas	2007	2007 NSCH	10–17	CDC-2000^2^	11.8	11.0	12.6	20.4	20.6	20.2	Singh et al., 2010 [20]
	USA, Utah	2007	2007 NSCH	10–17	CDC-2000^2^	11.7	12.1	11.2	11.4	14.7	7.9	Singh et al., 2010 [20]
	USA, Virginia	2007	2007 NSCH	10–17	CDC-2000^2^	15.8	16.1	15.4	15.2	16.6	13.9	Singh et al., 2010 [20]
	USA, Vermont	2007	2007 NSCH	10–17	CDC-2000^2^	13.8	16.4	11.1	12.9	17.0	8.4	Singh et al., 2010 [20]
	USA, Washington	2007	2007 NSCH	10–17	CDC-2000^2^	18.4	21.9	14.7	11.1	14.7	7.3	Singh et al., 2010 [20]
	USA, Wisconsin	2007	2007 NSCH	10–17	CDC-2000^2^	14.8	17.3	12.2	13.1	15.6	10.5	Singh et al., 2010 [20]
	USA, West Virginia	2007	2007 NSCH	10–17	CDC-2000^2^	16.6	16.9	16.4	18.9	21.8	15.7	Singh et al., 2010 [20]
	USA, Wyoming	2007	2007 NSCH	10–17	CDC-2000^2^	15.5	16.6	14.2	10.2	14.1	5.5	Singh et al., 2010 [20]
	Brazil, Pernambuco State	2006	GSHS	14–19	IOTF^1^	11.5	11.3	11.6	2.4	2.0	2.8	Tassitano et al., 2009 [40]

Asia	China, Hong Kong	2003-04	School-based survey	11–18	IOTF^1^	7.1	9.6	4.9	2.8	3.9	1.8	Ko et al., 2008 [41]
					CDC-2000^2^	8.3	11.3	5.8	4.1	6.0	2.4	
	India, Manipur	2005-06	School-based survey	12–19	IOTF^1^	4.4	4.1	4.7	0.7	1.0	0.4	Bishwalata et al., 2010 [42]
	Jordan, Irbid Governorate	2007	School-based survey	13–16	CDC-2000^2^	15.7	11.8	18.9	8.7	12.3	5.8	Abu Baker and Daradkeh, 2010 [43]

Europe	Denmark, Greater Copenhagen area and 3 municipalities outside the Capital Region	2007–09	School-based survey	14–16	IOTF^1^	14.0	15.2	12.9	11.2	14.1	8.2	Søren and Jo, 2010 [44]
	France, Aquitaine Region	2004-05	School-based survey	11–18	IOTF^1^	11.7	13.3	10.1	1.9	2.4	1.4	Thibault et al., 2010 [45]
	Greece, Crete	2005-06	School-based survey	10–12	IOTF^1^	28.0	30.0	27.0	13.0	15.0	10.0	Manios et al., 2011 [46]
	Hungary, Szeged and Szolnok regions	2005-06	School-based survey	11–16	IOTF^1^	16.8	17.9	15.7	6.6	7.9	5.2	Baráth et al., 2010 [47]
	Italy, North-West	2002	HBSC Study	11, 13, 16	IOTF^1^	—	18.3	7.1	—	2.5	1.1	Vieno et al., 2005 [34]
	Italy, North-East	2002	HBSC Study	11, 13, 16	IOTF^1^	—	16.5	11.7	—	0.8	1.5	Vieno et al., 2005 [34]
	Italy, Center	2002	HBSC Study	11, 13, 16	IOTF^1^	—	20.7	11.2	—	3.9	2.5	Vieno et al., 2005 [34]
	Italy, South	2002	HBSC Study	11, 13, 16	IOTF^1^	—	25.7	15.7	—	4.4	0.9	Vieno et al., 2005 [34]
	Italy, Islands	2002	HBSC Study	11, 13, 16	IOTF^1^	—	31.3	10.4	—	7.6	0.8	Vieno et al., 2005 [34]
	Italy, Sardinia	1999–2001	School-based survey	11–15	IOTF1	14.9	15.4	14.6	3.7	5.1	3.2	Velluzzi et al., 2007 [48]
	Italy, Sicily	1999–2001	Public school-based survey	11–15	CDC-2000^2^	18.3	18.8	17.8	11.8	15.1	8.5	Baratta et al., 2006 [49]
	Poland, Kujawsko-Pomorskie	2005	School based survey	13–15	IOTF^1^	10.7	12.0	9.5	1.4	1.6	1.3	Jodkowska et al., 2010 [50]
	Poland, Lubuskie	2005	School based survey	13–15	IOTF^1^	11.1	12.2	10.1	3.0	2.5	3.5	Jodkowska et al., 2010 [50]
	Poland, Malapolskie	2005	School based survey	13–15	IOTF^1^	12.7	12.8	12.6	1.6	1.9	1.3	Jodkowska et al., 2010 [50]
	Poland, Podlaskie	2005	School based survey	13–15	IOTF^1^	13.9	14.5	13.3	2.8	3.1	2.6	Jodkowska et al., 2010 [50]
	Poland, Pomorskie	2005	School based survey	13–15	IOTF^1^	13.7	13.4	13.9	2.1	2.0	2.2	Jodkowska et al., 2010 [50]
	Spain, Balearic Islands	2007-08	School-based survey	12–17	WHO-2007^3^	17.5	19.9	15.5	10.4	12.7	8.5	Bibiloni et al., 2010 [51]
	Spain, Grand Canary	2004-05	School-based survey	12–14	IOTF^1^	21.6	21.0	22.2	7.5	7.8	7.2	Henríquez Sánchez et al., 2008 [52]
	Switzerland, Canton of Vaud	2005-06	Public school-based survey	10–14	IOTF^1^	12.0	13.2	10.7	1.7	1.8	1.7	Lasserre et al., 2007 [53]
					CDC-2000^2^	10.7	11.9	9.4	3.6	4.2	3.0	
	Turkey, Edirne Province	2001	Two school-based surveys	12–17	IOTF^1^	10.9	11.3	10.6	1.9	1.6	2.1	Öner et al., 2004 [54]

^1^Overweight and obesity, all adolescents with BMI-for-age ≥25 kg/m^2^ and <30 kg/m^2^ and ≥30 kg/m^2^, respectively, according to the IOTF [6].

^2^Overweight and obesity, all adolescents with BMI-for-age ≥P85th and <P95th and ≥P95th, respectively, according to the CDC [5].

^3^Overweight and obesity, all adolescents with BMI-for-age ≥P85th and <P95th and ≥P97th, respectively, according to the WHO [7].

IOTF: International Obesity Task Force; CDC: Center for Disease Control and Prevention; WHO: World Health Organization; 2008 SA YRBS: 2008 South African National Youth Risk Behaviour; 2007 NSCH: National Survey of Children's Health; GSHS: Global School Based Student Health Survey; HBSC: Health Behaviour in School-aged Children.
